# Long‐term Engagement in a Digital Lifestyle Intervention to Reduce Dementia Risk in Older Adults

**DOI:** 10.1002/alz70860_105577

**Published:** 2025-12-23

**Authors:** Maria Bunyan, Philip Vassilev, Jennifer H Barnett, Jamie Kawadler, Sarah Bauermeister, John Gallacher, Ivan Koychev

**Affiliations:** ^1^ University of Oxford, Oxford, Oxford, United Kingdom; ^2^ Five Lives, London, United Kingdom; ^3^ Five Lives, London, London, United Kingdom; ^4^ University of Oxford, Oxford, Oxfordshire, United Kingdom; ^5^ Imperial College London, London, London, United Kingdom; ^6^ University of Oxford, Oxford, United Kingdom

## Abstract

**Background:**

The recent Lancet standing Commission estimated that 45% of dementia cases are potentially modifiable and suggested that lifestyle interventions may be suitable to address dementia risk. Delivering interventions digitally may offer accessibility, scalability and participant experience. Success of digital interventions will likely be determined by participant long‐term engagement. The following results are an update to a previously reported digital lifestyle intervention and describe the effect of demographic factors and inclusion of 1‐to‐1 clinician sessions on long‐term engagement.

**Method:**

We investigated a digital lifestyle intervention (mobile phone application Five Lives) in a study where it was paired with brief 1‐to‐1 virtual clinician sessions. Participants (*N* = 154) were aged 50‐69 with normal cognition and were randomised into four groups: control (received no intervention), application‐only group, and two groups with application + Virtual Clinic (receiving one or three sessions respectively). The digital intervention started with a CE‐marked dementia risk assessment, followed by a 12‐week digital coaching programme.

**Result:**

Engagement (number of application activities completed over 12 weeks) was predicted only by participant age, sex and baseline lifestyle score; with older, female participants with healthier lifestyles completing more activities. Long‐term engagement was variable with steady decline in the number of users active within each group from week 1 to week 12 (application‐only group week 1 = 82% and week 12 = 39%, application + 1 Virtual Clinic group week 1 = 82% and week 12 = 36%, and application + 3 Virtual Clinics week 1 = 90% and week 12 = 39%).

**Conclusion:**

Participant engagement suggests that the intervention is accessible to many participants. Long‐term application usage suggests the intervention is engaging for some participants. We found that it is participant baseline characteristics, such as age and pre‐intervention risk factors rather than the addition of 1‐to‐1 Virtual Clinic sessions that influenced long‐term engagement with the app. These findings warrant further investigation of digital lifestyle interventions for older adults at risk of dementia, particularly into the challenge of maintaining participant engagement. Future work should aim to identify what meaningful participant engagement is and what level of engagement facilitates sustained lifestyle change.

